# The Swiss health care atlas—relaunch in scale

**DOI:** 10.1007/s43999-022-00016-0

**Published:** 2023-01-12

**Authors:** Reto Jörg, Jonathan Zufferey, Oliver Zumbrunnen, Boris Kaiser, Stefan Essig, Marcel Zwahlen, Tobias Schoch, Marcel Widmer

**Affiliations:** 1 Swiss Health Observatory, Espace de L’Europe 10, 2010 Neuchâtel, Switzerland; 2 BSS Volkswirtschaftliche Beratung, Basel, Switzerland; 3 Interface Politikstudien, Forschung Beratung AG, Lucerne, Switzerland; 4 Departement of Health Sciences and Medicine, University of Lucerne, Lucerne, Switzerland; 5 Institute of Social and Preventive Medicine, University of Bern, Bern, Switzerland; 6 School of Business, University of Applied Sciences Northwestern Switzerland, Olten, Switzerland

**Keywords:** Atlas, Health care, Variation analysis, Small area analysis, Health service research

## Abstract

Inspired by the Dartmouth Atlas of Health Care, an early version of the Swiss Atlas of Health Care (SAHC) was released in 2017. The SAHC provides an intuitive visualization of regional variations of medical care delivery and thus allows for a broad diffusion of the contents. That is why the SAHC became widely accepted amongst health care stakeholders. In 2021, the relaunch of the SAHC was initiated to update as well as significantly expand the scope of measures depicted on the platform, also integrating indicators for outpatient care in order to better reflect the linkages between inpatient and outpatient health care provision. In the course of this relaunch, the statistical and technical aspects of the SAHC have been reviewed and updated. This paper presents the key aspects of the relaunch project and provides helpful insights for similar endeavors elsewhere.

## Introduction

**Fig. 1:**
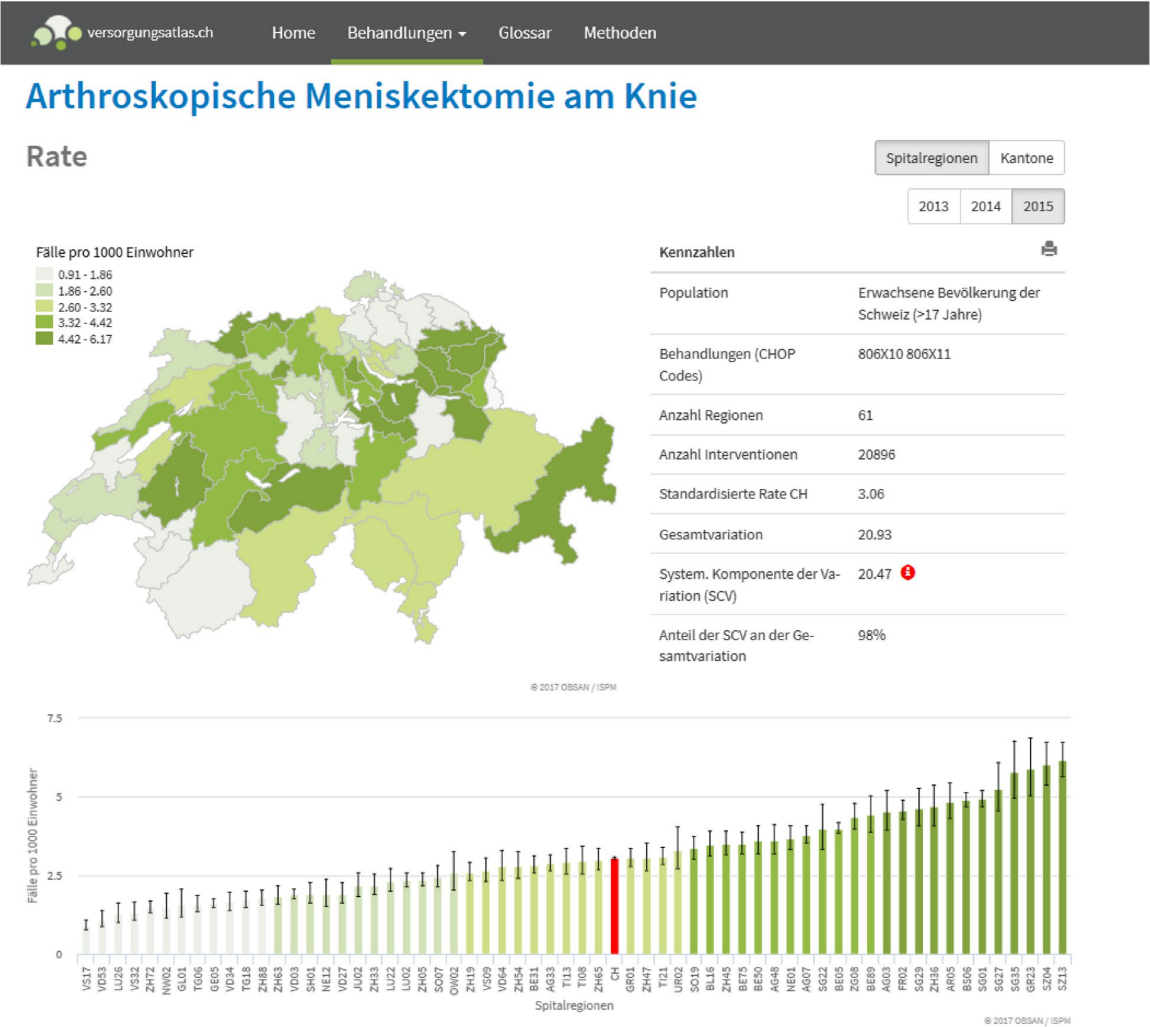
Example view of the early version of the Swiss Atlas of Health Care in 2017. This example shows the standardized rates for arthroscopic meniscectomy of the knee per HSA in 2015. Extracted from https://versorgungsatlas.ch/index.php/de/MENK/ (accessed 2022 September 20)

In June 2017, the Swiss Atlas of Health Care (SAHC) was published at www.versorgungsatlas.ch (see Fig. 1 for an example). At its early release, the SAHC included about 30 indicators on mainly surgical interventions. The indicators describe the frequency of these procedures by canton and hospital service area (HSA). The SAHC was a collaborative project involving the Institute of Social and Preventive Medicine (ISPM) at the University of Bern and the Swiss Health Observatory (Obsan). The work of John E. Wennberg and his colleagues [1, 2], including the Dartmouth Atlas of Health Care, served as an inspiration and a blueprint.

The SAHC provides easy-to-understand visualizations of regional variations in the provision of medical services. The interpretation of the maps and diagrams is intuitive and thus allows for a broad diffusion of the contents beyond the health research community. Furthermore, the SAHC can be utilized to identify fields of unwarranted variation of clinical practice. Practice variation is considered unwarranted when the variation is not explained by the incidence of illness or the preferences of patients [3]. In its capacity to pinpoint unwarranted variation, the SAHC serves both as a basis for political discourse about potential overuse or underuse and as a starting point for more in-depth research on possible drivers of high or low utilization.

The early SAHC was financed by the Gottfried and Julia Bangerter-Rhyner Foundation as part of the "Health Care Research" funding program of the Swiss Academy of Medical Sciences (SAMW). The atlas was very well received by relevant actors in health politics and public health administration and has since provided important impulses for the scientific discourse on the Swiss health care system [4–10]. Despite the high reputation, no long-term funding for a systematic updating and expansion of the SAHC could be found for a considerable time. For this reason, the Swiss Health Observatory, in collaboration with the Federal Office of Public Health (FOPH), has initiated a project to relaunch the health care atlas in 2021, with the atlas serving as a tool in the implementation of the federal council’s health policy strategy 2020–2030 [11] highlighting the role health data for efficient and optimal organization of health care and the impact of an inadequate allocation of health care resources on health costs and quality of care.

In subsequent sections, this article focuses on the various objectives associated with the relaunch of the SAHC. It will also provide information on the approaches and measures chosen to achieve these goals. The relaunch of the SAHC is an ongoing project. The release of the new SAHC is scheduled for the first quarter of 2023. This article is therefore to be understood as a preview and should ideally provide inspiring insights for other entities considering similar endeavors.

## Expanding scope of indicators

The early version of the SAHC comprises around 30 indicators, covering the field of surgical interventions in particular. All of the indicators concerned inpatient care. With the 2023 relaunch of the SAHC, the set of indicators will be significantly expanded. Currently, it is planned to publish around 115 indicators. The focus is on somatic care, with psychiatry and rehabilitation excluded for the time being. However, indicators for outpatient care (including primary care) will be integrated for the first time. Especially in light of the federal and cantonal health policy strategy to shift inpatient services increasingly to the outpatient sector (keyword "ambulant vor stationär", AVOS), it is crucial to examine the interfaces between inpatient and outpatient care. Regional differences in the frequency of certain interventions can often only be meaningfully assessed if inpatient and outpatient care are considered together. Examples include cataract surgery, hernia surgery, meniscectomy of the knee, implantation of a permanent cardiac pacemaker, crossectomy and stripping of varices, to only name a few. The expansion of the set of indicators also applies to the type of interventions considered. In addition to (surgical) interventions, diagnostic procedures as well as vaccinations and the dispensing of medications will be covered by the atlas.

**Fig. 2:**
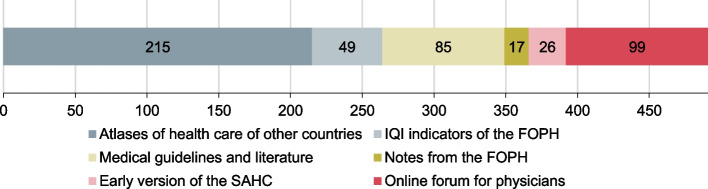
Source of the identified potential indicators (*n* = 491) for the SAHC relaunch

A multistep procedure was used to identify relevant indicators. In a first step, a list of potential indicators was compiled. Sources included the health care atlases of other countries, the quality indicators of Swiss acute care hospitals developed by the Federal Office of Public Health [12], medical guidelines and the scientific literature, as well as suggestions from various stakeholders. In an additional survey that was distributed via the medical societies, physicians were asked to share ideas for new indicators via an online forum. The online forum was password-protected, accessible around the clock, and did not require identification of participants. It allowed a wide range of physicians to be involved, from the grassroots to the executive committees of medical societies. In this first step, a total of 491 potential indicators were identified (Fig. 2).

**Fig. 3:**
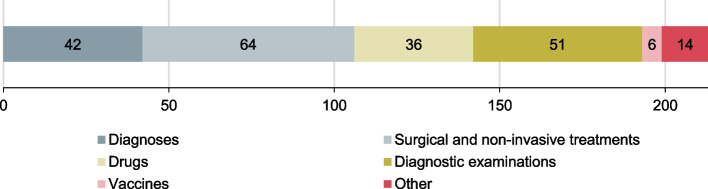
Identified potential indicators by category after feasibility check (*n* = 213)

In a second step, the feasibility was analyzed. Taking into account the data sources available in Switzerland, it was examined which indicator definitions could be used directly for the SAHC or could at least be adapted to be feasible with regard to the available data. Indicators often had to be simplified by ignoring the restriction to a patient group with a specific diagnosis, since information on diagnoses is not systematically collected for outpatient care in Switzerland. A total of 213 indicators passed the checks (see Fig. 3).

In a third step, a prioritization was performed involving the advisory board for this project, which included scientists as well as representatives of all relevant stakeholders. As a result, 171 potential indicators were pursued further.

In a fourth step, the definitions were developed for the indicators using the relevant coding and classification systems. Depending on the type of indicator, different classification systems are used: Swiss classification of medical procedures (Schweizerische Operationsklassifikation, CHOP)^1^ and International Classification of Diseases (ICD)^2^ for inpatient treatments, the fee-for-service system for outpatient medical services in Switzerland (TARMED)^3^ for outpatient treatments, the official list of laboratory analyses in mandatory health insurance (AL)^4^ and the Anatomical Therapeutic Chemical (ATC) Classification System^5^ for drugs. Table 1 contains illustrative examples of the respective classification systems and codes.

**Table 1:** Examples of codes according to tariff or classification system

Sector / indicator type	Classification system	Examples (translated from German)
Inpatient treatments	CHOP	45: Incision, excision and anastomosis of the bowel 45.41: Local excision of lesion or tissue in colon 45.41.1: Local excision of lesion or tissue in colon, endoscopic 45.41.13: Endoscopic submucosal dissection colon
	ICD	I: Diseases of the circulatory system I21: Acute myocardial infarction I21.0: Acute transmural myocardial infarction of the anterior wall
Outpatient treatments	TARMED Individual services TARMED Packages (lump sum compensation)	39: Imaging techniques (chapter) 39.5180: {MRI} Knee joint and/or lower leg (tariff item) 0001.0310.001: Mammography screening, cantonal program (rate type 002) 23.1301.00.05: Screening—Mammography—Flat rate per case (rate type 003)
Laboratory analyses	Analysis list (AL)	1363.01: Hemoglobin A1c
Pharmaceuticals (drugs / vaccines)	ATC	N02: Analgesics N02A: Opioids N02AA: Natural opium alkaloids N02AA01: Morphine

In a fifth and final step, the indicators were then validated and the definitive list of indicators was determined. Based on provisional data analyses and with the involvement of experts, the validity of the indicators and their relevance to health care policy were assessed.^6^ Table 2 shows the criteria for the validation in detail.

**Table 2:** Criteria for assessment of the final indicators

Criteria	Specification
Validity of the measurement	-Appropriateness and correctness of the indicator label -Appropriateness and correctness of the selected codes based on the classification and tariff systems -Unambiguity and reproducibility of the indicator -Adequacy and correctness of the method of calculation -Consistent definition of numerator and denominator; denominator describes the relevant population at-risk -Interregional comparability
Relevance to supply policy	-Any regional differences in the indicator are significant in terms of health care policy (strong variation would justify measures; variation is relevant for health care costs) -Case numbers are sufficiently high to meaningfully address regional differences corresponding to unwarranted variation

## Systematic updating of indicators

With the relaunch of the SAHC, the existing indicators will be redefined and updated with data from 2013 to 2021. Furthermore, the relaunch introduces a largely automated procedure for data processing and data handling, which includes data cleansing and preparation, calculation of the statistical key figures, and staging of the key figures for display on the platform. The automated procedure saves personnel resources and allows for quick updates when new data become available. Only this way the annual update of the SAHC can be ensured in the future, especially since less than 0.5 full time equivalents (FTE) per year are allocated to the regular operation of the platform. On top of that, Switzerland's multilingualism results in additional requirements with regard to the translation of content. For this purpose, an unambiguous interface has been established, which defines how the data from various sources have to be processed. The interface defines the input data structure as well as the relevant features (variables) to be specified for each indicator (Table 3).

**Table 3:** Examples of key features defining atlas indicators

Feature	Examples
*Definition of the numerator*	-Number of cases (e.g. hospitalizations) of patients over 65 years of age -Number of procedures (especially relevant for diagnostic imaging techniques, where several images can be performed per case) -etc
*Definition of the denominator (risk population)*	-Resident population over 65 -Inpatient births (e.g. caesarean sections) -Newborns -etc
*Classification system relevant for inclusion criteria*	-International Classification of Diseases (ICD) -Swiss surgical classification (CHOP) -Outpatient fee-for-service system (TARMED) -List of laboratory analyses (AL)
*Inclusion criterion: relevant codes*	For example, "treatment of femoral neck fracture" is defined using a combination of ICD and CHOP codes ICD: -"S72.1—Femoral neck fracture" CHOP: -"78.15.10—Open reduction of femoral neck fracture and proximal femur fracture with bone fixation by external fixator [L]" -"79.35.11—Open reduction of femoral neck fracture and proximal femur fracture with internal bone fixation [L]" -"79.15.20—Closed reduction of other femur fracture with internal bone fixation" -etc

## Embedding the SAHC in politics and science

The health care atlas is first and foremost considered a tool, which contains relevant information on regional variations in health care made accessible by intuitive visualizations. In order to have the intended impact when it comes to questions about future health care policy, the relevant stakeholders must use the SAHC (i.e. public administration at federal and cantonal level, physicians including medical societies, insurers, patients including patients advocacy groups, science community). Consequently, embedding the atlas in the relevant policy frameworks as well as within the academic community is a key aspect regarding the relaunch of the SAHC. Several measures were taken to achieve this goal.

First, a comprehensive advisory board was set up. In addition to members from the research community, the advisory board includes representatives of the cantons, service provider associations, health insurers, patient advocacy groups as well as the Federal Statistical Office (FSO) and the Federal Office of Public Health. The advisory board is supporting the relaunch in all stages of the project, beginning with the definition of relevant indicators and ending with the communication strategy in preparation for the release of the SAHC.

Second, in addition to the advisory board, further stakeholders were involved in the determination of indicators in order to ensure that the information presented in the SAHC is valid and relevant to health care policy. This concerns in particular the relevant divisions and sections of the FOPH, which of course belong to the key target groups of the SAHC. This was done with the aim of introducing the SAHC as an integral part of existing processes within the FOPH. For example, it is envisioned that in the future the monitoring of radiation exposure will be based on indicators from the SAHC, depicting the frequency of exposure to ionizing radiation in medicine (including from X-rays, CT scans, and dental and nuclear imaging).

Third, in order to incorporate the SAHC into the academic environment, a summer school is scheduled for 2023. The purpose of this summer school is to establish the analysis of regional variations as a branch of health services research in Switzerland. With reference to the SAHC, concepts such as "small area analysis", "unwarranted variation" and "evidence-based health care policy" will be introduced to a new generation of PhD and post-doc students.

## Methodology review

In the first version of the atlas, rates were indirectly standardized with respect to demographic characteristics (age and sex). Quantification of regional variation was based on the systematic component of variation (SCV) of McPherson and colleagues [13]. The SCV enjoys great popularity in the field of small area variation analysis, but it also has some disadvantages [14].

With the relaunch, the following four statistical aspects are shown in the atlas: 1) directly standardized rates (incl. confidence intervals) to facilitate comparisons over time, 2) the SCV, 3) an Empirical Bayes (EB) estimate of the variance that complements the SCV, and 4) a ratio of high versus low rates across small areas based upon the work of Coory and Gibberd [15]. The ratio is defined as the quintile ratio (QR) – i.e., the ratio of the 80% to the 20% quantile – of the predicted rates. Prediction refers to the EB predictions under a Poisson-Gamma model [16, 17], which also defines the EB measure of variance. The QR is an intuitively appealing measure that does not suffer from the statistical problems that exist with the extremal quotient of the crude rates [14, 15].

## Technical Upgrade

The existing SAHC was operated with a content management system (CMS), which has since been discontinued. As a result, a redevelopment of the web platform has been required. A new web application was developed to support the highly automated process for creating and updating the data. The application is based on JavaScript frameworks (most important: react.js and next.js). Furthermore, in the existing atlas the Highcharts JavaScript Library was used to generate the graphics. In the new release, the graphics will be created using Apache ECharts.^7^ Finally, the SAHC will be integrated into the Obsan website and the design will be adapted accordingly.

## Automatic derivation of HSA regions

A key point of the SAHC is the type of regionalization applied. While health care utilization rates of treatment are traditionally mapped using administrative regions (cantons, districts, etc.), the SAHC relies on Hospital Service Areas (HSAs). The HSAs capture the catchment areas of each hospital. This allows geographic variations to be described in the context of the particular care delivery systems.

For the early version of the SAHC, the HSA regions were determined in a laborious, largely manual process [18]. For the relaunch, the R package ‘HSAr’ is used to derive the HSAs.^8^ The package was developed within the National Research Programme *Smarter Health Care* (NRP 74) and is freely available [5]. The package can be used, both, to account for effective patterns of utilization based on patient flows and to ensure spatial contiguity of resulting HSAs. In the SAHC, the HSAs were determined in such a way that within a region at least 40% of all somatic patients are treated in hospitals within that region. The choice of a relatively low location index (below 50%) is motivated by the aim of mapping peripheral regions that only have one or more hospitals delivering primary care. It should be noted that in Switzerland, since 2012, the free choice of hospital has also been established for patients with basic health insurance. Because of this, and because of the relatively short distances between different agglomerations in Switzerland, it is not surprising that HSAs are only partially self-contained. The SAHC comprises 74 HSAs,^9^ which were additionally validated with a panel of experts. From the main HSAs, different hospital referral regions (HRR) have been derived, mapping the utilization of rarer treatments and medical procedures (e.g. cardiac surgery) that have a different geography than somatic care as a whole.

## Limitations of the existing data sources

Regarding the inpatient sector, the SAHC relies primarily on the microdata drawn from the Medical Statistics of Hospitals (MS)^10^ of the Federal Statistical Office. By contrast, indicators on outpatient care are based on aggregated claims data from health insurers. The latter are taken from the pooled databases (“Tarifpool” and “Medicube”)^11^ of SASIS AG, a data service provider of the Swiss health insurers. Other data sources can be added for individual indicators.

The technical challenges (e.g. data quality issues and lack of data standards), ethical-legal challenges (e.g. legal uncertainty and health data ownership issues), sociocultural challenges (e.g. declining trust in institutions that collect health data) and procedural challenges (e.g. lack of oversight of health data sources) of access to health data in Switzerland have been well documented lately [19]. In Switzerland, many health care data are routinely collected and stored in the process of clinical care or to meet regulatory requirements (e.g. in terms of billing). However, the data is often stored in unconnected, inconsistent data silos, each with their own acquisition, transport, storage and validation processes. Importantly, there is also a strong difference between data collection in outpatient versus inpatient health care. While in the inpatient sector, detailed case-level microdata on services and costs are available, similar microdata from outpatient care are largely lacking. Moreover, in the inpatient sector, diagnoses are coded according to the ICD, whereas no systematic coding of diagnoses or reasons for encounter is used in the outpatient sector [20]. Furthermore, there is no Unique Personal Identifier (UPI) in the Swiss health care system, which would allow to easily combine (pseudonymized) data on the same person across different databases and sectors [21].

With respect to the aforementioned challenges to health data access, there are two primary limitations for the SAHC:

1. Indicators that include outpatient care services cannot include diagnostic information because diagnostic information is not collected within the outpatient setting (e.g. HbA1c measurements for diabetes patients, imaging of the lower spine in response to unspecific low back pain).
2. Indicators that include a combination of services along the clinical pathway requiring the linking of different data sources at the patient level are hardly feasible. This refers to indicators that take into account different treatment episodes in different settings (e.g. pre-operative chest x-ray, Aspirin prescription within 2 weeks after acute myocardial infarction (AMI), outpatient follow-up treatments after inpatient surgery)

Even if these limitations are significant, the available data can still be used to generate important information for the Swiss health care system. Moreover, by demonstrating the benefits of already accessible data the SAHC is also intended to contribute to the discussion on health data accessibility.

## Conclusion

The SAHC is an important and widely accepted tool for monitoring the health care delivery system in Switzerland. The visually intuitive and interactive design allows a wide range of users to engage with the data and moreover fosters in-depth health care research. The project to relaunch the SAHC aims to provide a sustainable foundation for the SAHC in both technical and financial terms. This paper presents the key elements and highlights some of the main challenges of the relaunch project and thus aims to provide helpful insights for similar endeavors elsewhere. Due to the small size of Switzerland and the associated limitations with regard to the available resources combined with the requirements due to multilingualism, it is essential to establish an efficient and largely automated workflow in order to provide the SAHC with the required data in regular intervals. At the same time, the relaunch is also a matter of scaling the idea and integrating plenty of new indicators. Here, the inclusion of the outpatient sector is crucial, considering the shift to outpatient care in the Swiss healthcare system. To increase the impact of the SAHC across the entire Swiss health care system, all relevant stakeholders including representatives of public administration at federal and cantonal level, medical societies, insurers, patient advocacy groups and the science community were involved in the relaunch project. A high level of participation was thus already achieved within the course of the project and, together with the stakeholders, it was already possible to outline how the atlas can be practically embedded in public governance processes.

## Footnotes

1 See https://www.bfs.admin.ch/bfs/de/home/statistiken/gesundheit/nomenklaturen/medkk/instrumente-medizinische-kodierung.html

2 See https://www.bfs.admin.ch/bfs/de/home/statistiken/gesundheit/nomenklaturen/medkk/instrumente-medizinische-kodierung.html

3 See www.tarmed-browser.ch

4 The analysis list (AL) contains those analyses that are provided by medical laboratories and covered in accordance with Swiss Federal Law on Compulsory Health Care, see https://www.bag.admin.ch/bag/de/home/versicherungen/krankenversicherung/krankenversicherung-leistungen-tarife/Analysenliste.html

5 The ATC is a drug classification system that classifies the active ingredients of drugs according to the organ or system on which they act and their therapeutic, pharmacological and chemical properties. It is controlled by the World Health Organization Collaborating Centre for Drug Statistics Methodology (WHOCC), see https://www.whocc.no/atc/structure_and_principles/

6 The criteria used are adapted versions of the evaluation criteria for quality indicators in health care by the Institute for Quality Assurance and Transparency in Health Care, Berlin, Germany, see https://iqtig.org/, and the Institute for Applied Quality Promotion and Research in Health Care, Göttingen, Germany, see https://www.aqua-institut.de/

7 See https://echarts.apache.org/en/index.html

8 See https://github.com/aghaynes/HSAr

9 It should be noticed that around 40% of the HSAs have locations index above 60%, around 30% of the HSAs have locations index between 50 and 60%, and around 30% of the HSAs have locations index between 40 and 50%.

10 See www.bfs.admin.ch/bfs/de/home/statistiken/gesundheit/erhebungen/ms

11 See www.sasis.ch
